# Peripheral-central interplay for fatiguing unresisted repetitive movements: a study using muscle ischaemia and M1 neuromodulation

**DOI:** 10.1038/s41598-020-80743-x

**Published:** 2021-01-22

**Authors:** Elena Madinabeitia-Mancebo, Antonio Madrid, Antonio Oliviero, Javier Cudeiro, Pablo Arias

**Affiliations:** 1 Universidade da Coruña, Neuroscience and Motor Control Group (NEUROcom), Department of Sport and Physical Education, Department of Physiotherapy, Medicine and Biomedical Sciences, INEF-Galicia, Institute of Biomedical Research of A Coruña, A Coruña, Spain; 2 FENNSI Group, Hospital Nacional de Paraplejicos, SESCAM, Toledo, Spain; 3 Hospital Los Madroños, Brunete, Madrid, Spain; 4 Centro de Estimulación Cerebral de Galicia, A Coruña, Spain

**Keywords:** Neuroscience, Motor control, Neural circuits, Sensorimotor processing

## Abstract

Maximal-rate rhythmic repetitive movements cannot be sustained for very long, even if unresisted. Peripheral and central mechanisms of fatigue, such as the slowing of muscle relaxation and an increase in M1-GABA_b_ inhibition, act alongside the reduction of maximal execution rates. However, maximal muscle force appears unaffected, and it is unknown whether the increased excitability of M1 GABAergic interneurons is an adaptation to the waning of muscle contractility in these movements. Here, we observed increased M1 GABA_b_ inhibition at the end of 30 s of a maximal-rate finger-tapping (FT) task that caused fatigue and muscle slowdown in a sample of 19 healthy participants. The former recovered a few seconds after FT ended, regardless of whether muscle ischaemia was used to keep the muscle slowed down. Therefore, the increased excitability of M1-GABA_b_ circuits does not appear to be mediated by afferent feedback from the muscle. In the same subjects, continuous (inhibitory) and intermittent (excitatory) theta-burst stimulation (TBS) was used to modulate M1 excitability and to understand the underlying central mechanisms within the motor cortex. The effect produced by TBS on M1 excitability did not affect FT performance. We conclude that fatigue during brief, maximal-rate unresisted repetitive movements has supraspinal components, with origins upstream of the motor cortex.

## Introduction

Maximal-rate rhythmic repetitive movements (RRMs) cannot be sustained for very long, even when the movements are unresisted, and it is known that the slowing of muscle relaxation is a major peripheral mechanism of RRM fatigue^1,2^. However, there is also growing evidence for a neural origin of fatigue during these types of movements^3–5^, and their characteristics clearly differ from those expressed during fatiguing isometric contractions performed with the same body segment and for an equivalent period of time^6,7^.

During maximal-rate finger tapping (FT), even for periods as short as 30 s, the tapping frequency reduces rapidly, the muscle slows, and the excitability of spinal motor neurons and M1 inhibitory interneurons (operating through GABA_b_ receptors) increases^6–8^. Movement deterioration during fatiguing FT is similar to that observed during unresisted RRM executed by other body segments^5^. This observation suggests that fatigue may impact central structures engaged in rhythm formation and stresses the importance of understanding the physiological mechanism of fatigue in many situations, such as activities of daily living, sports, and the care of neurological patients. Likewise, because fatigue deteriorates different unresisted RRM in similar manners^5^, the use of FT is supported as a model for the study of some components of fatigue in these movements. On the other hand, non-invasive brain stimulation techniques offer the possibility of modifying cortical excitability in an attempt to avoid fatigue development during these types of movements, but the protocol to be used (to increase or decrease excitability) would depend on whether the increased excitability of M1 inhibitory interneurons^6–8^ is a central adaptation to waning muscle contractility^8^.

Central adaptations to the fatigued muscle state have been studied in different ways, one of which is to maintain muscle ischaemia^9–13^. In the fatigued muscle, there is an increase in the concentrations of metabolites (such as H^+^, ADP, or P_i_) and a decrease in ATP turnover, which may cause the contractile properties of the muscle to slow and may simultaneously stimulate different muscle nociceptors and chemoreceptors. These receptors (polymodal free nerve endings^14,15^) give rise to small afferents (type III and IV) that project to different levels of the central nervous system^15–17^, such as the inhibitory interneurons at the spinal cord^18^. Contractility slowdown remains if blood flow is occluded using a cuff^19^, which is thought to impair metabolic restoration in the muscle and keep the free nerve endings stimulated. Under these conditions, the contractile properties of the muscle do not recover until ischaemia is relieved^19^.

To shed light on the fundamental mechanisms of fatigue, we test for peripheral-central interplay in the context of fatiguing unresisted repetitive movements. We employed the FT test, a well-known test for studying rhythmicity control at the clinical level^20^. In contrast to resisted repetitive actions such as cycling^21^, unresisted repetitive movements require low levels of muscle force, and the effects of fatigue in these tasks do not compromise central drive to the muscle or muscle force generation, at least for short-duration tasks^8,22^.

**Figure 1:**
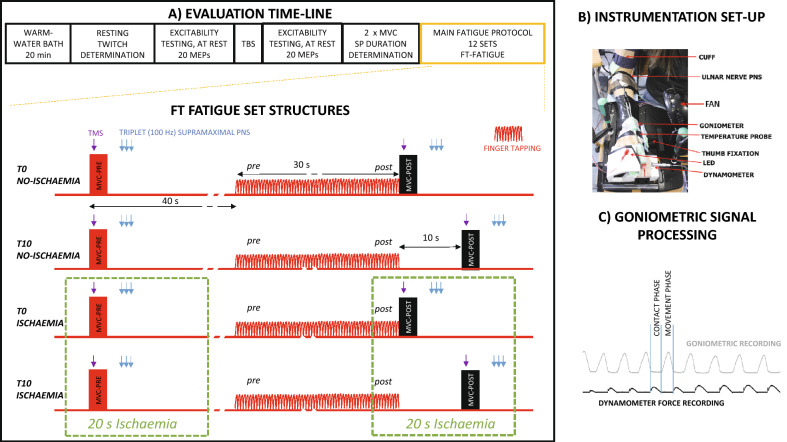
Protocols used for FT fatigue testing. (**A**) Evaluation timeline and structure of FT fatigue testing conditions: after soaking the hand and forearm in a warm-water bath for 20 min, we determined the intensity of percutaneous electrical stimulation needed to acquire muscle resting twitches. Then, using paired-pulse TMS over M1, we tested corticospinal excitability with motor evoked potential (MEP) amplitudes and long intracortical inhibition (LICI). Next, we applied M1 theta-burst stimulation (TBS; intermittent [iTBS], continuous [cTBS] and sham [shamTBS] across different sessions, on different days), and MEP and LICI were retested. Each day, after receiving TBS, subjects performed two brief MVCs (with 3 min rest between) to determine the M1-TMS intensity to be used in the fatigue protocol; this was the intensity needed to produce SP durations of ≈ 200 ms in the two MVCs. Subsequently, subjects performed 12 FT sets (four different set-conditions sequentially repeated three times) with 3 min rest between every set. The *T0 NO-ISCHAEMIA* condition started with a brief MVC (PRE) in response to a light-emitting diode (LED) turning on. At the MVC plateau, muscle force was recorded, and TMS was delivered to acquire SP; 500 ms after the TMS pulse, the LED turned off as a cue to stop the MVC. Three seconds later, and with the muscle at rest, we applied percutaneous nerve stimulation (a triplet at supramaximal intensity) to produce a resting muscle twitch. With this twitch, we calculated the HRT of the muscle offline. Next, 40 s after the sequence of events had started, subjects executed FT at the maximal possible rate for 30 s. Immediately after FT (with no gap), subjects performed another brief MVC (POST), and stimulations were applied as before. The remaining conditions were similar to those described above, with small differences. For *T10 NO-ISCHAEMIA*, the MVC POST FT (and stimulation) started after a delay of 10 s following the end of FT; during the delay, subjects remained at rest. For the ischaemia conditions (*T0 ISCHAEMIA* and *T10 ISCHAEMIA*), the sets were as above but had 20 s of muscle ischaemia at the time of stimulation testing. (**B**) Instrumentation and set-up: the different units are indicated by arrows in the panel. The apparatus was worn by the subjects immediately after removing their arms from the water bath. (**C**) Signal processing: this method was used to determine the features of the tapping profile, as described in detail in the “Methods” section.

In this work, our aim was to test whether the increased GABA_b_ inhibition in M1^6–8^ and the impairment in muscle contractility^8^ observed during maximal-rate unresisted RRM were related. Therefore, we recorded M1 GABA inhibition by means of transcranial magnetic stimulation-induced silent periods (TMS-SPs)^23^ during maximal voluntary contraction (MVC) and muscle contractility slowdown by means of the half-relaxation time (HRT)^1,2,8^ immediately after a fatiguing FT task (without resting time). However, in some sets, the SP and HRT were recorded after a period of 10 s of rest from FT, which is sufficient time to allow complete SP recovery^24^. To ensure that the contractile properties of the muscle remained slow at the time of recording (i.e., 10 s after FT), we tested these variables while imposing muscle ischaemia in some of the sets^10,12,25,26^ (Fig. 1 briefly summarizes the protocol, further detailed in the “Methods”).

As per our hypothesis, if SP recovers within 10 s of stopping FT as the muscle is slowing down, the increase in SP observed at the very end of FT^6–8^ would not be representative of a central adaptation to the muscle state. Conversely, if SP remains enlarged after 10 s from the end of FT during muscle ischaemia, the increase in the excitability of M1-GABA interneurons (in this case, inhibition mediated by GABA_b_ receptors) would likely be due to a supraspinal adaptation to a less functional muscle.

Once such central-peripheral interplay is defined, the modulation of M1 excitability might clarify some of the mechanisms involved in this interaction. It might also contribute to reduced fatigue during the task. For these reasons, the protocol outlined above was performed in combination with neuromodulation techniques in a randomized order in three different sessions: continuous and intermittent theta-burst transcranial magnetic stimulation of M1 (cTBS and iTBS, respectively) and shamTBS. cTBS and iTBS are neuromodulation techniques generally accepted to decrease and increase excitability, respectively^27,28^.

As per our second hypothesis, if the shamTBS session shows that the increased GABA_b_ inhibition in M1 is a central adaptation to a less functional muscle, increasing M1 excitability with TBS will not reduce fatigue development. In fact, it might have detrimental effects. In such a case, decreasing M1 excitability might produce superior FT performance.

## Results

Table 1 shows the normalizing scores of the outcome variables in the 3 sessions (n = 19 completed all sessions). These scores did not differ significantly across sessions and correspond to the units of the y-axes in the graphs.

**Table 1:** Normalizing scores (mean across participants and standard error of the mean [SE]) for different sessions.

	Maximal FT rate (Hertz, Hz)	Maximal possible active index finger ROM (°)	FDI SP duration at PRE (milliseconds, ms)	HRT at PRE (milliseconds, ms)	MVC force (kilograms, kg)
shamTBS	6.79 SE 0.21	46.85 SE 1.97	226.37 SE 8.34	65.22 SE 2.32	8.71 SE 0.51
cTBS	6.75 SE 0.19	49.17 SE 1.08	222.84 SE 6.82	65.11 SE 2.96	8.36 SE 0.53
iTBS	6.78 SE 0.23	50.23 SE 1.26	230.22 SE 7.11	64.41 SE 2.75	8.24 SE 0.53

Please see “Methods” for description.

*FT* finger tapping, *ROM* range of motion amplitude, *FDI SP* first dorsal interosseous silent period, *HRT* half-relaxation time, *MVC* maximal voluntary contraction, *SE* standard error of the mean.

### Effect of slowing muscle relaxation on increased M1-GABA_b_ inhibition after fatiguing FT

**Figure 2:**
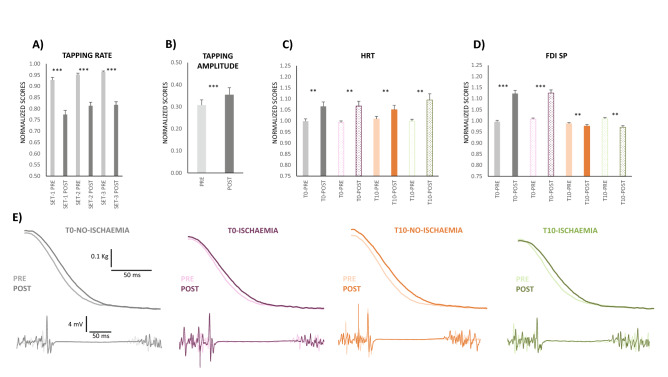
Expression of fatigue in the absence of M1 neuromodulation (shamTBS session). (**A**) FT rate and (**B**) FT ROM amplitude variables are shown by pooling delays and ischaemia since these factors had no significant effects. The maximal FT rate decreased significantly in 30 s (PRE vs. POST) for all sets and conditions (both delay and ischaemia); with set progression, the maximal rate increased both at PRE and POST (p < 0.001, asterisk omitted). The FT ROM amplitude at POST was larger than PRE for all sets and conditions. (**C**) HRT and (**D**) FDI SP are split by delays and ischaemia conditions for visual comparison (solid bars = no ischaemia; textured bars = ischaemia); HRT showed similar changes for all of them. The HRT (**C**) increased significantly from PRE to POST, and this increase was not different across the delays (T0 and T10), ischaemia levels and set progression. The FDI SP (**D**) increase from PRE to POST was significant only for T0. For T10, SP was significantly reduced at POST. The effects of FT on SP were not different for the two ischaemia conditions. (**E**) Representative recordings of FDI SP and relaxation phase of the resting twitch from the same individual in different ischaemia and delay conditions. **p ≤ 0.01, ***p ≤ 0.001. The y-axis unit score values are shown in Table 1.

To test our first hypothesis, we analysed the shamTBS session. The maximal rate of FT decreased in 30 s (F_1,18_ = 153.4, p < 0.001_TIME_) and the PRE-POST decrement was ≈ 15%. However, from set to set, the FT rate increased (F_2,36_ = 13.0_ε=0.6_, p < 0.001_SET_); the change was small (≈ 4%) and likely reflected learning effects (Fig. 2A).

The range of motion amplitude during FT also increased at the end of the task (F_1,18_ = 15.1, p = 0.001_TIME,_ Fig. 2B). The change in magnitude was small (≈ 5%) and was present in all sets. The remaining main effects and interactions were not significant.

At the peripheral level*,* muscle contractility slowed POST FT (F_1,18_ = 12.6, p = 0.002_TIME_); this is expressed by an ≈ 7% increase in HRT in all sets (the remaining factors and interactions were not significant). The absence of significant interactions of the TIME factor (PRE-POST) with other factors implied that the muscle slowing observed at POST-T0 remained at T10 for all conditions (Fig. 2C). Importantly, the change in HRT was not influenced by changes in muscle temperature, as temperature was experimentally controlled and constant for the duration of the session [33.4 °C, standard error of the mean (SE) 1.3] (main effects and interactions were not significant).

M1-GABA_b_ inhibition (i.e., FDI SP duration) changed at the very end of FT (F_1,18_ = 47.7, p < 0.001_TIME_), but did so differently for the two delays tested (F_1,18_ = 104.8, p < 0.001_TIME X DELAY_). Post hoc analyses showed that SP tested immediately after FT (T0) increased from PRE to POST by ≈ 12% (p < 0.001), but not if tested after 10 s of rest (POST-T10), where it was 2.5% smaller than PRE (p = 0.002) (Fig. 2D). In all conditions and for set progression, the SP duration increased slightly but significantly (≈ 3.5% from the beginning to the end of the protocol, F_2,36_ = 10.1, p < 0.001_SET_; effect omitted in graphs). Figure 2E shows individual recordings of FDI SP and HRT on a PRE-POST basis for the different conditions.

In summary, increased GABA_b_-mediated inhibition in M1 was evident immediately after FT, but SP recovered after 10 s of rest when the muscle remained slowed. Therefore, the increased SP duration observed right at the end of maximal-rate FT was not a response to reduced muscle contractility (i.e., increased HRT) observed at the end of the task.

### Effect of real TBS on fatigue expressions

**Figure 3:**
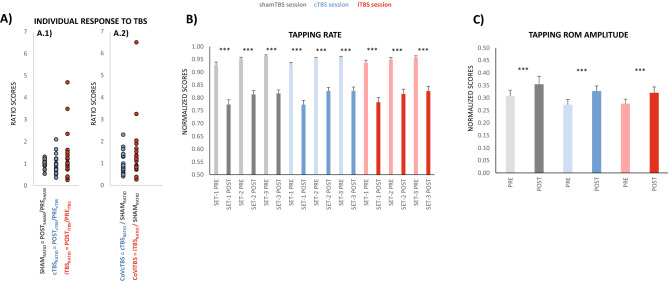
Effects of M1-TBS on fatigue expressions for maximal FT rate and FT ROM. (**A**) Individual responses to TBS; (**A1**) shows the individual scores of the ratio calculated with MEP amplitude PRE and POST TBS for each session (SHAM_RATIO_, cTBS_RATIO_ and iTBS_RATIO_). MEP amplitude scores (mean across participants) at PRE did not differ between sessions (ANOVA p = 0.33; 1.8 mV SE 0.3, 1.8 mV SE 0.3, 1.4 mV SE 0.2, respectively). (**A2**) shows the individual scores of the grand ratios (cTBS_RATIO_/SHAM_RATIO_ and iTBS_RATIO_/SHAM_RATIO_). These scores indicate each individual response to cTBS and iTBS compared to shamTBS and were introduced in ANOVA as covariables to test the aftereffects of TBS on fatigue responses. (**B**) Maximal FT rate and (**C**) FT ROM amplitude at PRE and POST after different TBS protocols; cTBS and iTBS did not produce different responses compared to shamTBS for any variable, and this effect was not different for individual responses to TBS. **p ≤ 0.01, ***p ≤ 0.001. The y-axis unit score values for (**B**) and (**C**) are shown in Table 1.

Figure 3A shows individual changes in cortical excitability produced by cTBS and iTBS compared to shamTBS*,* tested immediately before FT execution. We used these scores as covariates (CoVcTBS and CoViTBS) to evaluate the individuals’ aftereffects of TBS on fatigue profiles.

For the tapping rate, TBS produced no changes in subject performance (Fig. 3B). When we analysed responses after cTBS and compared them with those obtained after shamTBS, the factor STIM (including sham vs. cTBS levels) did not interact significantly with any other factors at the sample level or with the covariate CoVcTBS (at the individual level). The same applies for the analyses of sham and iTBS levels of factor STIM, with CoViTBS as a covariable. Likewise, the maximal tapping rates acquired along any of the testing points and sets did not differ across the sessions (Table 1).

Therefore, the changes in M1 excitability produced by TBS did not change either the maximal FT rate obtained at any of the protocol testing points (Table 1) or fatigue development during the 30 s.

**Figure 4:**
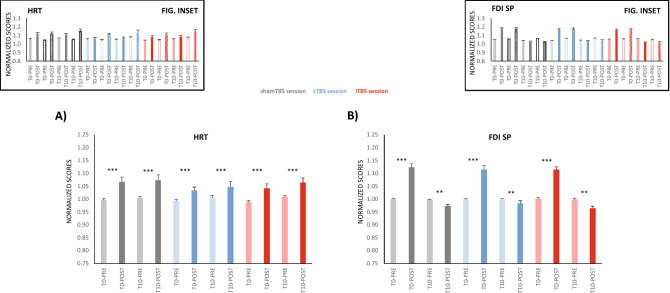
Effects of M1-TBS on HRT and FDI SP duration. (**A**) HRT and (**B**) FDI SP show results by pooling those from the ischaemia condition since ischaemia as a main effect was not significant and did not have any significant interactions with other factors. For both variables, the responses of cTBS and iTBS did not differ significantly from those observed for shamTBS. HRT increased significantly at POST (both T0 and T10). SP was significantly increased POST FT only for T0 and reduced at T10, and this did not differ across different TBS sessions. The insets indicate responses for HRT and SP before and after FT in the delay and ischaemia conditions for the three TBS protocols (solid bars = no ischaemia, textured bars = ischaemia); asterisks denoting significance were omitted as the effects were not different from the pooled responses in (**A**) and (**B**). **p ≤ 0.01, ***p ≤ 0.001. The y-axis unit score values are shown in Table 1.

The tapping ROM amplitude (Fig. 3C), HRT and FDI SP (Fig. 4A,B) followed similar patterns in their responses to TBS protocols. None of the variables changed significantly with cTBS or iTBS compared to *sham*TBS protocols at the group level or individually (when considering CoVcTBS and CoViTBS covariates).

**Figure 5:**
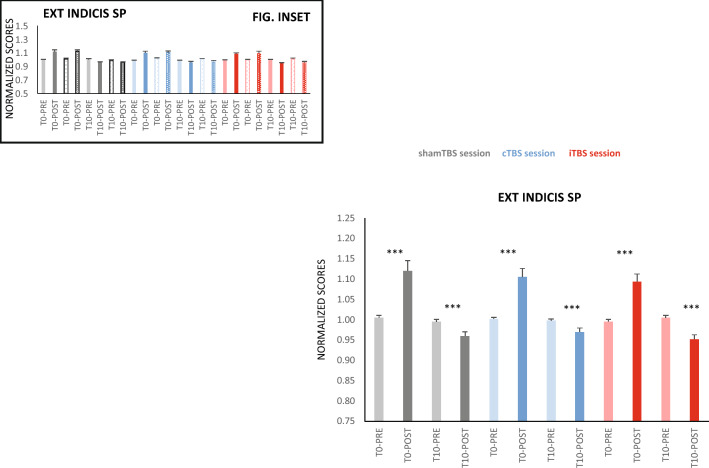
Effects of M1-TBS on extensor indicis SP duration. Ischaemia levels were pooled for extensor indicis (EXT-IND) SP, since they were not significantly different and had no significant interactions with other factors. Responses to cTBS and iTBS did not differ significantly from those observed after shamTBS. EXT-IND SP was significantly larger POST (vs. PRE) FT only for T0 but smaller for T10, and this did not differ across different TBS sessions. Figure insets indicate responses of EXT-IND SP before and after FT in delay and ischaemia conditions for the three TBS protocols (solid bars = no ischaemia, textured bars = ischaemia). The asterisks denoting significance were omitted because they were not significantly different from the pooled response in the main plot. **p ≤ 0.01, ***p ≤ 0.001. The y-axis unit scores are equivalent to PRE SP durations for the different sessions (207.7 ms SE 9.3 for shamTBS; 205.9 ms SE 7.3 for cTBS; and 212.7 ms SE 8.3 for iTBS), which did not differ significantly.

Our set-up and experimental design was aimed to study the main FT flexor (i.e., TMS *hotspot* corresponded to *first dorsal interosseous* cortical representation, and HRT was tested during flexion force). As additional data, we also recorded electromyography activity from the *extensor indicis* muscle (EXT-IND). The responses of the EXT-IND SP followed the same pattern than FDI SP in the three sessions (Fig. 5).

In summary, the changes in M1 excitability produced by TBS did not change the tapping profiles during the fatiguing FT task.

### Aftereffects of TBS on MVC muscle force

**Figure 6:**
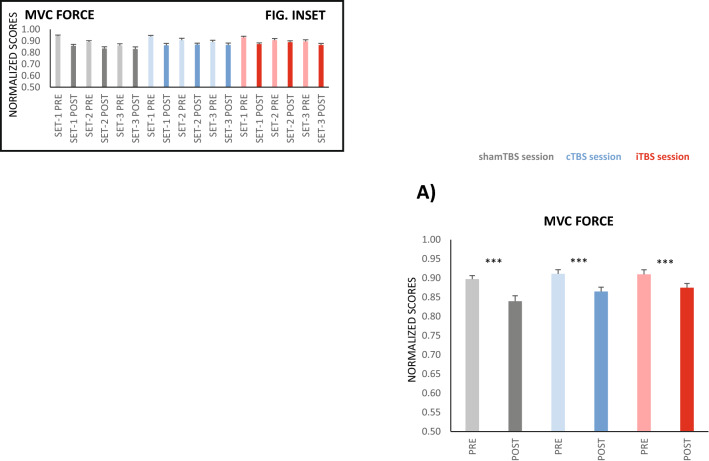
Effects of M1-TBS on MVC force. MVC change (PRE vs. POST FT) by pooling sets (**A**) and with set progression (inset); (**A**) shows the results on a PRE-POST basis by pooling sets; the drop in MVC force POST FT was significantly smaller in the iTBS session than in the shamTBS session, but PRE-POST changes remained significant for all stimulation modes, as denoted by asterisks. In the three sessions conducted, MVC force decreased significantly with set progression, but the reduction was larger at PRE than at POST. The inset depicts the significantly different PRE-POST change with set progression and the significant change with set (asterisk omitted for clarity). ***p ≤ 0.001. The y-axis unit score values are shown in Table 1.

Although our primary objective was not to study TBS aftereffects on muscle force, we performed MVCs before and after FT to allow the recording of M1 SPs, and muscle force was analysed. In the shamTBS session, muscle force decreased from PRE to POST FT and progressively after each set (F_1,18_ = 36.6, p < 0.001_TIME_ and F_2,36_ = 9.9, p < 0.001_SET_) (Fig. 6, inset). Notably, the drop in muscle force across sets was more pronounced before FT (at PRE) than at POST (F_2,36_ = 12.4, p < 0.001_TIME X SET_) (Fig. 6 inset)_._ This response in the sham session was not different from the response observed in the cTBS session (i.e., the factor STIM [cTBS vs. shamTBS] and covariate CoVcTBS did not interact significantly with any other factor).

Conversely, this was not the case for iTBS, where the MVC force drop from PRE to POST FT differed from that observed for shamTBS (F_1,17_ = 11.7, p = 0.003_STIM×TIME_). The effect was detected at the population level and was not dependent on the manner in which iTBS changed M1 excitability in individuals, as CoViTBS had no significant interactions.

Whereas the PRE-POST force loss was 5.7% for shamTBS (and 4.6% for cTBS), it was reduced by only 3.5% for the iTBS session (Fig. 6).

In summary, and in response to our second hypothesis, the aftereffects of TBS did not change the profile of fatigue during FT. This was observed in both the whole sample of participants and considering individual changes in corticospinal excitability after cTBS and iTBS. We also performed analyses considering TBS aftereffects on long intracortical inhibition (LICI; see “Methods”), but the results did not show any change from those reported above.

## Discussion

Fatigue produced by unresisted RRM at the maximal rate has central and peripheral manifestations. When the maximal rate of FT drops at the end of 30 s, the contractility of the muscle slows^8^ and M1 SP, not the spinal SP, enlarges^6–8^. However, the central drive to the muscle remains unchanged^8^.

In response to the first hypothesis proposed in this study, we determined that the increased inhibitory activity (GABA_b_) at the level of M1 is not a response to waning muscle contractility. Therefore, the increased cortical SP observed at the end of fatiguing FT has a central origin.

The expressions of tapping fatigue observed in this study are consistent with previous works with similar tasks^6–8^, showing null effects of our shamTBS protocol on behaviour. The maximal FT rate drop was ≈ 15% after 30 s of tapping^6–8^, the HRT increased by ≈ 7%^8^, and M1 silent periods enlarged by 12%^6,8^. However, 10 s of rest after FT allowed the SP to recover, even when the muscle remained slowed down.

Aside from the above results, there are three other observations from our study that deserve some commentary. First, the HRT 10 s after FT was similar in both the presence and absence of muscle ischaemia*.* This implies that these peripheral mechanisms of fatigue recover much more slowly^19^ than central expressions^24^. Second, when 10 s of rest was allowed after FT, the SP duration was smaller than PRE; thus, it not only recovered to baseline levels but also reduced even further. Despite the small magnitude of change (2.5%), this phenomenon suggests an active mechanism of recovery, either by disinhibition or by modification of excitation. Third, the *first dorsal interosseous* SP pattern of response was also observed for SP recorded from the *extensor indicis* muscle. Although our set-up and design aimed at testing the main FT flexor (i.e., TMS hotspot was on *first dorsal interosseous* cortical representation, and HRT was tested on flexion force), we also recorded electromyography (EMG) activity from the *extensor indicis*, whose SP duration behaved the same as that of the *first dorsal interosseous*. This observation (similar response in antagonistic muscles) reinforces the concept that increased M1 GABA_b_ inhibition originates at central levels and not from afferent feedback; the latter is mainly homologous but can also affect antagonist muscles^29^ (or even act trans-segmentally^30^). It is worth mentioning that an antagonistic mechanism of inhibition is absent in our work because both the *first dorsal interosseous* and *extensor indicis* SPs recovered in the presence of muscle slowdown.

On the other hand, the results of maximal voluntary force indicated that it dropped at the very end of FT and with set progression (in agreement with previous works that did not use neuromodulation techniques^6,7^). However, we previously showed that the decline in force was not produced by FT^6–8^ but was due to the repeated execution of MVC during the session (slow fatigue development did not affect cortical SP^6–8^).

We observed a small but significant increase in SP duration from set to set, a profile very different from the large increase in SP seen at the end of fatiguing FT. Because SP recovery to pre-fatigue values is very rapid (less than 10 s^24^) and increased intracortical inhibition is also associated with motor learning, we believe that the small increase in SP duration observed from set to set in our study is produced by motor learning. In line with this possibility, maximal tapping frequency also increased across sets (another sign of learning). Learning in fast rate tapping has been previously described within sessions^31^, and this fast learning modulates M1 excitability^31,32^. It is known that different inhibitory processes contribute to TMS-SP duration (GABA_a_ for short SPs and GABA_b_ for long SPs)^23,33^; for this reason, we propose that in our study, the effects of two different GABAergic networks can be identified: one is responsible for fatigue within sets after 30 s FT, and the other is related to learning across sets.

In summary, our results during the sham TBS session suggest that fatigue generation during unresisted RRM originates from two unrelated central and peripheral mechanisms. The increased GABA_b_ inhibition of M1 is not a response to the increased muscle relaxation slowdown observed at the end of FT. Provided that fatigue characteristics of rate drop for unresisted RRM are similar for very different effectors (such as finger and foot)^5^, our observations might represent a generalized profile of fatigue for these movements.

### Effects of changing M1 excitability with TBS on fatigue induced by maximal-rate FT

Once a supraspinal origin of fatigue is defined, the aftereffects of TBS on M1 excitability would allow a more detailed understanding of its regulatory mechanisms. cTBS produced no effect on the tested variables, and behaviour during this session did not differ from that observed in the sham TBS session. This was not the case for the iTBS session; however, the iTBS impact did not focus on FT behaviour but on the force levels developed immediately after it.

iTBS modulates M1 excitability by acting on interneuronal populations engaged in the generation of late I-waves, recorded from the descending volleys towards spinal motor neurons, in response to cortical stimulation^34^. It is known that these late I-waves are dependent, at least in part, on GABA_b_ intracortical inhibition^23,35^, which enlarges upon FT fatigue^6–8^. Therefore, we might expect a clear aftereffect of iTBS upon FT fatigue, assuming that supraspinal fatigue originates at M1. However, we did not observe any changes upon FT fatigue in the whole sample of participants, even accounting for individual responses in M1 excitability to iTBS. These results support the idea that SP extension with fatigue originates at supraspinal structures other than M1, which might direct or indirectly target the motor cortex. This possibility is also supported by the fact that the aftereffects of the other active TBS protocol in our study, cTBS, did not change the FT profiles either. The supraspinal origin of fatigue outside M1 also appears to be a possibility for tasks different to unresisted RRM, for instance for sustained or repeated isometric contractions^36,37^.

Previous studies applied neuromodulation techniques on repetitive movements but focussed on resisted contractions such as cycling (see Machado et al.^38^ for review). In such studies, techniques such as anodal transcranial direct current stimulation and iTBS on M1 allowed either greater motor output generation or longer time to task failure^21,39,40^. iTBS on M1 might be suitable for treating changes in force during fatigue; in fact, we also observed a positive aftereffect of iTBS in preventing loss of force. However, neither waning of muscle force^4,8,22^ nor central drive to the muscle^8^ are characteristic mechanisms of fatigue resulting from unresisted rhythmic movements, due to which iTBS might not be an effective solution for unresisted RRM fatigue. Alternatively, a central disruption in rhythmicity control might be the underlying cause (or an associated mechanism) of such a form of neural fatigue, as previously published results point to an increased co-activation of antagonistic muscles^5,22^ and a breakdown of M1 surround inhibition^5^ in such scenarios. Our observations of maximal tapping rate loss and the slight but significant increase in ROM amplitude might be the consequences of ineffective activation sequencing of index flexors and extensors (i.e., fast changes from extension to flexion). This implies that the central origins of fatigue emerge from a supraspinal network other than M1 deeply engaged in rhythmic formation (basal ganglia, for instance), even when the primary motor cortex might be one of the final nodes. Finally, a previous work indicated that an excitatory protocol (paired-TMS at I-wave interval) combined with motor practice reduced the drop in tapping rate along 10 s of fast FT; however, in this case, tapping was not maximal^31^, as clearly shown in a complementary experiment presented in Supplementary Fig. 1.

## Conclusions

Unresisted repetitive movements at the maximal possible rate produce fatigue, expressed as a fast drop in the maximal movement rate and increased excitability of inhibitory GABA_b_-dependent M1 intracortical circuits. This process is not mediated by afferent feedback from the muscle, but it originates at supraspinal levels.

The absence of any aftereffects of TBS (either cTBS or iTBS) on M1 for such behavioural and neurophysiological landmarks suggests that the increased M1 inhibition with FT fatigue development is generated by supraspinal inputs targeting M1, perhaps arising from subcortical structures that are engaged in rhythm formation.

## Methods

The protocols were bound by the Declaration of Helsinki and approved by the University of A Coruña Ethics Committee. Participants signed informed consent forms.

### Participants

Nineteen of 23 healthy right-handed volunteers completed three scheduled sessions (10 males and 9 females; mean age 23.9 SEM 1.4); the 4 who refused to participate after the first session reported discomfort produced by the electric stimulation used in the protocol as cause of withdrawal. Sessions were identical in all respects, except for the kind of cortical stimulation applied prior to task execution. Cortical stimulation, as described below, included three types of TBS to modulate M1 excitability (shamTBS, cTBS and iTBS). The order of sessions was randomized across participants, with a minimum inter-session delay of 10 days.

### Fatiguing FT testing protocol

Behavioural evaluation during the FT task was similar to a previously described protocol^8^ (Fig. 1A). The upper dominant limb of the subjects was adapted to a 3D system^8^, which secured the forearm and hand (with the thumb fixed in abduction) but left the index finger free to move around the metacarpophalangeal joint. A force load metre (P200 Biometrics Ltd.) was attached to the system underneath the tip of the index finger, and an electrogoniometer (S100 Biometrics Ltd.) monitored the range of motion of the index metacarpophalangeal joint (Fig. 1B).

A single session comprised 12 FT execution sets, with 3 min of rest in between sets. Every set included the following sequence of events:

- The set started with the execution of an MVC of the index finger against the force load metre in response to an LED turning on. The index finger applied force in the direction of flexion of the metacarpophalangeal joint.
- During the MVC, a single TMS pulse was applied on the left M1, and the SP was recorded from the EMG activity. We monitored EMG activity from the superficial head of the *first dorsal interosseous* muscle, which is the main index metacarpophalangeal joint flexor when the thumb is fixed in abduction (other muscles were also monitored, some of them were for objectives unrelated to this study). The TMS pulse to induce SP was delivered at the time of the MVC peak by using a previously described and validated algorithm^8^; 500 ms after the TMS pulse delivery, the LED turned off, prompting subjects to stop the MVC and relax.
- With the muscle at rest, 3 s after the LED turned off, we applied percutaneous electric stimulation of the ulnar nerve (a 100 Hz triplet) to produce a resting muscle twitch, whose force was recorded. This sequence of events comprised PRE testing before FT (Fig. 1A).
- Forty seconds after the LED turned on (≈ 38 s of rest after the end of MVC), a low tone auditory cue was used as a signal to start FT at the fastest possible rate for 30 s. The instructions given to the subjects were as follows: “Tap with your index finger on the force sensor at your maximal possible rate at all times from the very beginning of the task, indicated by an auditory cue, to the end of the task indicated by a second cue; always tap as fast as possible”. No instructions were given about FT ROM amplitude or tapping force on the plate.
- After tapping for 30 s, we performed testing and stimulation as previously described for PRE. However, this POST testing was performed at two different times: (i) immediately after finger tapping, without a time gap from FT to MVC^8^ (T0), and (ii) after a 10 s period of rest (T10) from FT to MVC (Fig. 1A). Thus, for T0, subjects changed from FT to MVC without any time lag in response to the illumination of the LED. For T10, a non-startle auditory cue at the end of the 30 s of FT prompted subjects to stop tapping and rest for 10 s before performing the MVC.

Half of the 12 sets were T0, and the other half were T10, presented in a counterbalanced order. Subjects had no problems in understanding the instructions provided at the start of the protocol to execute the T0 and T10 sets. They were informed of the nature of the upcoming set (T0 or T10) during the 3 min rest periods, immediately prior to the start of the set. There were no mistakes during execution.

### Ischaemia

During half of the T0 and T10 sets, PRE and POST testing were performed under ischaemic muscle conditions (Fig. 1A). Muscle ischaemia was produced by inflating a cuff (at 300 mmHg) placed around the arm immediately above the right elbow (Fig. 1B). The cuff was automatically inflated using an electric pump (at PRE, inflation started 4 s before the LED cue for MVC execution). Inflation at 300 mmHg was acquired in < 0.6 s and sustained for 20 s; it finished 24 s before the start of FT.

At POST, ischaemia started in all sets (either T0 or T10) at the 26th second of the 30 s FT task duration and was sustained for 20 s. Therefore, for the POST-10 testing, ischaemia ended ≈ 2 s after recording the resting muscle twitch induced by percutaneous nerve stimulation (see below).

In summary, in each set, we studied one of these four conditions: T0_NO-ISCHAEMIA_, T0_ISCHAEMIA_, T10_NO-ISCHAEMIA_ and T10_ISCHAEMIA_. The presentation order of these four conditions in the first four sets was counterbalanced across participants, and this order was repeated for each subject in sets number 5–8 and 9–12. For a given subject, the sequence was repeated in the same order for all sessions (shamTBS, cTBS, or iTBS).

### TMS

TMS was performed in different ways to test and modulate M1 excitability during each session. We used single and paired pulses and *TBS*, following this sequence (Fig. 1A):

i. Evaluation of M1 excitability at rest using paired-pulse protocols (detailed below).
ii. M1 excitability modulation with TBS*,* as described below.
iii. M1 excitability was retested at rest with paired-pulse protocols (i. and iii. were meant to determine individuals’ response to *TBS* prior to FT execution).
iv. M1 excitability was tested during the FT fatiguing protocol using single pulse TMS.

### M1-excitability evaluation protocol

In all the above cases, M1 excitability was tested with a MagPro X100 with the MagOption system connected to a figure-eight coil (MC-B70) placed over the *hot-spot* of the FDI muscle, which was determined before the *theta-burst* and fatiguing protocols. The coil position was marked on the scalp with a soft pen for monitoring position during the session.

For testing M1 excitability, TMS pulses were monophasic-waveform and the coil oriented to induce currents in M1 in a postero-anterior (PA) direction.

- *Protocol for testing M1 excitability before the FT fatiguing protocol* At rest, we tested corticospinal excitability and LICI (*GABA*_*b*_ activity); this was the PRE value. Excitability was checked again in the same way after M1 was modulated with TBS (POST; see description below, and Fig. 1A). This evaluation was meant to determine the individual response of each subject to TBS given the variable responses to this form of stimulation from subject to subject^41^. We delivered 20 TMS paired pulses at PRE TBS (each pulse in the pair had the same intensity). TMS intensity was set to produce a Test-MEP (i.e., the first MEP of the pair) with an approximate amplitude of 1–1.5 mV; this first MEP amplitude was used to evaluate corticospinal excitability. The inter-stimulus interval (ISI) between pulses was explored between 100 and 200 ms and was selected to obtain a conditioned MEP (i.e., second MEP) of ≈ 50% of the Test-MEP, mean ISI interval across participants was 160.5 ms SE 4.9, range [130–200 ms]. The ISI determined in the first session was repeated in the other two sessions. The ratio (2nd MEP/1st MEP amplitude) was used to obtain M1 LICI (which is GABA_b_ dependent^23^).
  Likewise, another 20 TMS pairs were delivered POST theta burst, with the same intensity as PRE.
- *Testing M1 GABA*_*b*_* activity during the main FT fatigue protocol, the silent period* Single pulses of TMS were delivered during MVCs (PRE and POST FT) at the moment the MVC peaked and force plateau started. The timing was controlled using a previously described and validated algorithm by computing the slope of the force exerted on the dynamometer^8^. A video and data showing how the algorithm functions are available^8^. TMS intensity was set to induce an SP duration of ≈ 200 ms in the fresh muscle^6,8^, and the intensity was kept constant during the rest of the session.

### Modulation of M1 excitability

We applied TBS to modulate M1 excitability just before FT (Fig. 1A), with the subjects at rest. We used the MagPro X100 system with a refrigerated figure-eight coil (Cool-B65) positioned over the *hotspot* of the FDI muscle (the position was recalculated as this coil size was different). Biphasic waveform pulses were delivered to induce currents in the brain in a postero-anterior/antero-posterior direction. Stimulation intensity was set at 80% of the active motor threshold determined, immediately before TBS, with the Cool-B65 coil and biphasic waveform on the *first dorsal interosseous hotspot* for each session. A total of 600 pulses were delivered according to the iTBS and cTBS protocols described by Huang et al.^42^. For the shamTBS session, an unplugged figure-eight coil was placed over the *first dorsal interosseous* hotspot with another (fully operative) coil tilted at an angle of 90° over the first coil^43,44^.

### Electric percutaneous nerve stimulation

To test contractile muscle properties during the FT fatiguing protocol, we applied percutaneous nerve stimulation on the ulnar nerve at the elbow with a Digitimer DS7AH electric stimulator (0.2 ms pulse width). The anode was placed lateral to the medial epicondyle of the humerus along the post-condylar groove; the cathode was ≈ 2 cm distal to the anode on the nerve. Stimulation was a 100 Hz triplet at supramaximal intensity, which was 150% of the intensity needed to obtain maximal compound muscle action potential in the *first dorsal interosseous* muscle^8^. This intensity was calculated at the beginning of each session before any action was performed by the subjects (Fig. 1A). The force of the muscle twitch produced by the triplet was recorded by the dynamometer and used offline to calculate the *half relaxation time*. The *half relaxation time* reflects the contractile properties of the muscle and is defined as the time period from peak force to 50% of the peak force, calculated during the relaxation phase of the muscle twitch induced by the triplet. Since *half relaxation time* depends on muscle temperature^19,45^, we monitored and controlled muscle temperature for all the experimental sessions.

### Muscle temperature monitoring and stabilization

At the beginning of every experimental session, the participant’s dominant hand and forearm were immersed in a bath of warm water (≈ 33 °C) for 20 min^45^. During the fatiguing protocol, a thin temperature probe (3 mm tip YSI Temperature Probe, YSI Inc., Dayton, OH, US) was placed just beneath the adhesive part of the surface EMG electrode on the FDI muscle. The probe connected to an amplifier (CITER, CIBERTEC Ltd.) that sampled temperature (at 0.1 Hz) and sent the reading to a control unit (CED1401mkII, unit-1). Temperature was maintained at a stable level for the entire duration of the FT protocol by means of a flow of cool or warm air, as needed (Fig. 1B shows one of the fans used, placed aside for visibility of other components). Muscle temperature was estimated based on previous studies^45^: muscle temperature = 1.02 skin temperature + 0.89.

### Variable acquisition

MEPs, EMG activity and SP evoked by stimulation were recorded with surface electrodes placed in the belly-tendon position with Digitimer Ltd. D360 amplifiers (gain 250, bandpass 3–3000 Hz). The *first dorsal interosseous* was the target muscle whose activity was recorded and sent to a CED Ltd. 1401mkII unit 1 (sampling at 10 kHz and storing signals); we also recorded activity from the *extensor indicis*. Finger movements were monitored using an electrogoniometer (S100) and finger force using a dynamometer (P200), both connected to a K800 amplifier (Biometrics Ltd.) and sent to CED1401mkII unit 1 for sampling (10 kHz) and storage. In parallel, the force signal was sent to another CED1401mkII (unit 2) run with Sequencer. Sequencer (sampling at 100 kHz) ran an algorithm (previously described in detail and validated^8^) to trigger TMS at MVC peak plateaus and percutaneous nerve stimulation in the resting muscle; the transistor-transistor logic (TTL) triggers from CED1401mkII unit 2 to the stimulators were also sent to CED1401mkII unit 1 as TTL markers to facilitate offline data processing.

### Signal processing and analysed variables

Fatigue was defined as waning of the maximal tapping frequency during task execution^4–8,22,31,46^. To analyse fatigue, we tested differences in the variables defined below at PRE (i.e., acquired during the first MVC and, for FT frequency and ROM amplitude, during the first 5 s of the FT task) and at POST. For POST, we considered the second MVC and the last 5 s of FT for each set (Fig. 1A). Customized MATLAB (MathWorks Ltd.) programs were used to extract the following variables, in accordance with previous studies^6–8,47^:

- *Finger-tapping rate* The median frequency of all events included in the first 5 s (PRE) and the last 5 s (POST) of FT, with tapping cycles obtained from the force sensor^8^. For each session and subject, the tapping rates at all PRE and POST tested time points were normalised to the maximal FT rate obtained across all testing time points and sets^6–8^.
- *FT ROM amplitude* For each tapping cycle, the median value of the goniometric recording during the contact phase of tapping was subtracted from the peak value during the movement phase (Fig. 1C). The median of all cycles within the same 5 s time window, as above, was defined for PRE and POST. For each session and subject, the ROM amplitude for the PRE and POST time points of all sets was normalised to the ROM amplitude recorded during a full range extension movement, which was performed before the FT fatiguing protocol^6–8^.
- *SP duration* This was defined as the time from TMS trigger to recovery of EMG activity after the period of EMG silence induced by stimulation, as determined previously^6–8^. For each subject, the SP duration at the different time points was normalised to the mean of all SPs recorded at PRE across conditions for a given session, in agreement with previous studies^6–8^.
- *Half relaxation time and muscle temperature* HRT was defined as the time needed for returning from 100% (peak force) to 50% in the relaxation phase of the twitch force produced by triple percutaneous nerve stimulation, recorded with the muscle at rest after the MVCs. Within this period, we also extracted the median muscle temperature from thermometer recordings.

Apart from the variables cited above that were essential to test our hypotheses, we also analysed the MVC force, defined as the median force value in a 50 ms time window just prior to TMS (delivered at MVC peak^8^).

### Statistical analyses

To test our first hypothesis on the interaction of waning muscle contractility (i.e., increase in HRT) and increased M1-GABA_b_ inhibition (SP duration) during fatigue, we performed an analysis of variance with repeated measures (ANOVA_RM_) with the aforementioned variables in the shamTBS session. The factors of analyses were TIME (PRE, POST), SET (first, second, third), ISCHAEMIA (with, without), and DELAY (T0, T10).

Based on the abovementioned analyses, the second hypothesis was that if the increased M1 inhibition observed with fatigue is an adaptation to less functional muscle contractility, increasing excitability with TBS would worsen fatigue expressions on FT, and decreasing excitability might provide some benefits, and vice-versa. To check this possibility, we used another ANOVA_RM_, this time adding the kind of stimulation as a factor of analysis (STIM) and aftereffect of TBS on each individual’s M1 excitability, tested just prior to FT fatiguing, as a covariable. The covariate was included because cTBS can increase excitability rather than produce inhibition in many subjects, a paradoxical response also observed for iTBS^41^. Thus, two independent ANOVAs were performed with factor STIM (shamTBS, cTBS) or STIM (shamTBS, iTBS), each including a covariate.

The covariates were as follows:

- CoVcTBS for ANOVA with (shamTBS, cTBS)
- CoViTBS for ANOVA with (shamTBS, iTBS)

Individual scores for CoVcTBS were calculated as follows: at rest and before the fatigue protocol, we took the median of the 20 test MEPs acquired either PRE or POST TBS (Fig. 1A). With these scores from the cTBS and shamTBS sessions, we calculated the ratio of POST:PRE for each session. Subsequently, we calculated the CoVcTBS as the grand ratio (ratio of the ratios):

$${\text{CoVcTBS}} = \, \left( {{\text{POST}}_{{{\text{cTBS}}}} /{\text{ PRE}}_{{{\text{cTBS}}}} } \right)/\left( {{\text{POST}}_{{{\text{shamTBS}}}} /{\text{ PRE}}_{{{\text{shamTBS}}}} } \right).$$

If CoVcTBS = 1, this means that the change from PRE to POST was equivalent in the two sessions; CoVcTBS < 1 means that cTBS was inhibitory compared to shamTBS, and CoVcTBS > 1 means that cTBS was excitatory compared to shamTBS. CoViTBS was calculated in a similar manner.

In another set of analyses, we also considered the changes produced by cTBS and iTBS on LICI to evaluate the effects of TBS on the FT profile. We proceeded as above to define CoV_LICI_cTBS and CoV_LICI_iTBS. However, we restricted these analyses to those subjects for whom the PRE-POST TBS changes in the first MEP obtained with the paired-TMS pulses were not larger than 30% within sessions. In this way, we could assess how cTBS and iTBS changed LICI compared to shamTBS, with similar amplitudes of 1st MEPs. The analyses with CoV_LICI_cTBS included 10 participants and with CoV_LICI_iTBS 9 participants. However, the results were similar to those obtained with the whole sample of participants considering CoVcTBS and CoViTBS. Therefore, we omitted the report of CoV_LICI_ analyses in the results section.

In all figures, the graph values represent the mean across subjects along with the SEM. The normality of the distributions was checked with the one-sample Kolmogorov–Smirnov test, and the assumption of sphericity was tested using the Mauchly test in preparation for ANOVA; if sphericity was violated, the degrees of freedom were corrected with Greenhouse–Geisser coefficients. A Bonferroni correction was applied for post hoc comparisons within different levels of the factors. A result was considered to be significant when p ≤ 0.01^48^.

## Supplementary Information

Supplementary Figure 1.

## Data Availability

The data that support the findings of this study are available from the corresponding author upon reasonable request.
