# Supplementary material for: Peripheral-central interplay for fatiguing unresisted repetitive movements: a study using muscle ischaemia and M1 neuromodulation
Source: Sci Rep. 2021 Jan 22;11:2075. doi: 10.1038/s41598-020-80743-x (PMC7822864; doi:10.1038/s41598-020-80743-x)
Supplement: Supplementary file 1 — Supplementary Figure 1. [file 41598_2020_80743_MOESM1_ESM.pdf]

SUPPLEMENTARY FIG. 1

MAXIMAL POSSIBLE RATE (MPR): "Tap with your index on the force sensor at your maximal possible rate at all times from the very beginning of the task, indicated by an auditory cue, to the end of the task indicated by a second cue, tap always as fast as possible. The task lasts 10 seconds"

MAXIMAL SUSTAINABLE RATE (MSR): "Move your index finger as fast as you can, while trying to maintain both rate and amplitude for the whole 10 s duration; set the movement amplitude at a range that you feel comfortable with"

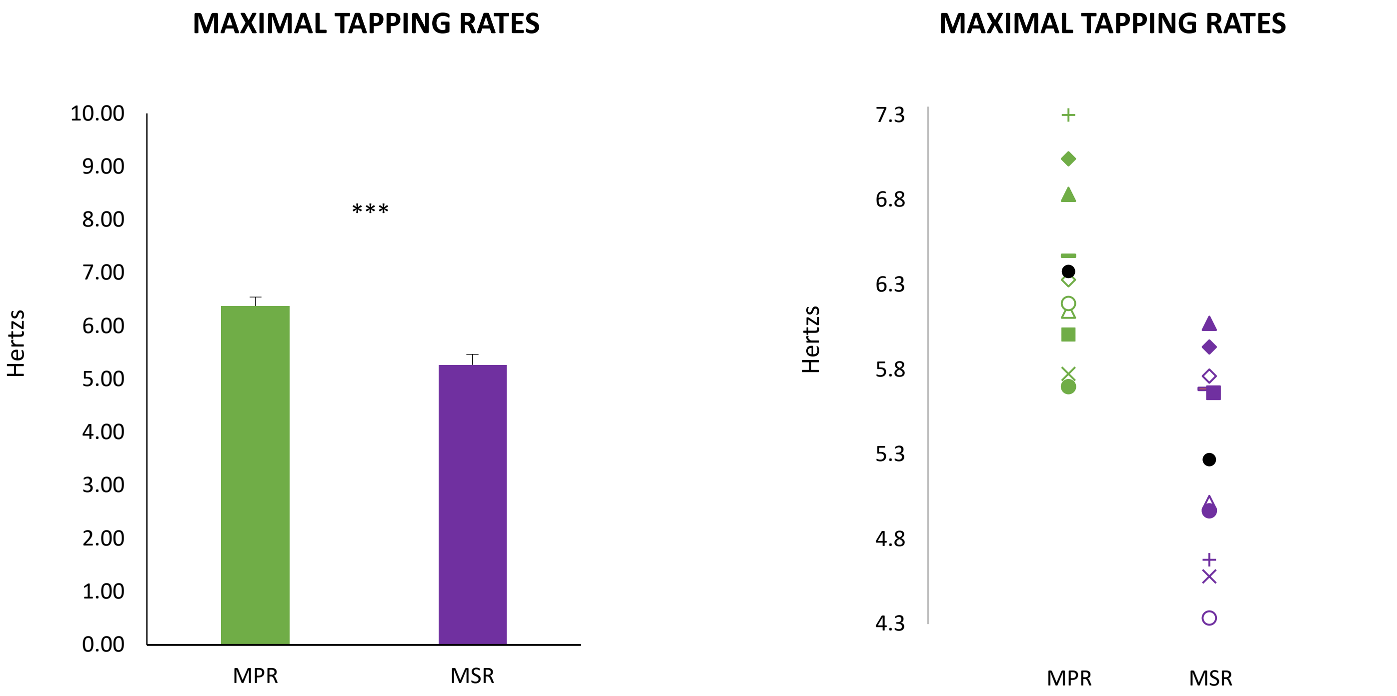

Supplementary Figure 1. Effect of verbal Instructions on FT Profiles

Ten healthy right-handed volunteers participated [5 women, aged between 19-27 years] in this complementary experiment. None of them took part in the main experiments to avoid possible carry-over learning effects. With the same instrumentation and set up as the main experiment, participants were asked to tap with their index finger of the dominant hand for 10 s[31]. They executed 6 sets with 5 min rest periods between sets. In three of the sets, they were given the following instructions: *"Tap with your index on the force sensor at your maximal possible rate at all times from the very beginning of the task, indicated by an auditory cue, to the end of the task indicated by a second cue, tap always as fast as possible. The task lasts 10 seconds"*; no instructions were given about FT ROM amplitude or tapping force on the plate. This tapping mode (the same as in our main experiments) is referred to as the maximal possible rate (MPR). In the other three sets, participants were given the following instructions: "*Move your index finger as fast as you can, while trying to maintain both rate and amplitude for the whole 10-s duration; set the movement amplitude at a range that you feel comfortable with*", as in previous work[31]. We referred to this tapping mode as the maximal sustainable rate (MSR). The tapping modes alternated in order of execution, and half of the subjects started the session with MPR. From the initial 2 s of the 10 s task[31], we extracted the median tapping rates for all tapping cycles included in this time window for all the different sets and conditions. Scores from three sets of each condition (MPR and MSR) were averaged and compared using Student's t-test (after checking for normality). Tapping rates were significantly different between conditions ($t_9 = 5.2$, $p < 0.001$), and MSR was ≈ 20% slower than MPR. **A)** Mean and SE across subjects. **B)** Individual responses in purple and green; black spots are mean values across participants.
